# Air pollution, cardiovascular endpoints and susceptibility by stress and material resources: a systematic review of the evidence

**DOI:** 10.1186/s12940-017-0270-0

**Published:** 2017-06-14

**Authors:** Christina H. Fuller, Karla R. Feeser, Jeremy A. Sarnat, Marie S. O’Neill

**Affiliations:** 1 Division of Environmental Health, School of Public Health, Georgia State University, Atlanta, GA USA; 2 Department of Environmental Health, Rollins School of Public Health, Emory University, Atlanta, GA USA; 3 Departments of Environmental Health Sciences and Epidemiology, School of Public Health, University of Michigan, Ann Arbor, MI USA

**Keywords:** Particulate matter, Air pollution, Traffic, Susceptibility, Cardiovascular, Stress, Socioeconomic, Effect modification

## Abstract

**Background and Methods:**

Evidence shows that both the physical and social environments play a role in the development of cardiovascular disease. The purpose of this systematic review is two-fold: First, we summarize research from the past 12 years from the growing number of studies focused on effect modification of the relationships between air pollution and cardiovascular disease (CVD) outcomes by socioeconomic position (SEP) and; second, we identify research gaps throughout the published literature on this topic and opportunities for addressing these gaps in future study designs.

**Results:**

We identified 30 articles that examined the modifying effects of either material resources or psychosocial stress (both related to SEP) on associations between short and long-term air pollution exposure and CVD endpoints. Although 18 articles identified at least one interaction between an air pollutant and material resource indicator, 11 others did not. Support for susceptibility to air pollution by psychosocial stress was weaker; however, only three articles tested this hypothesis. Further studies are warranted to investigate how air pollution and SEP together may influence CVD.

**Conclusions:**

We recommend that such research include thorough assessment of air pollution and SEP correlations, including spatial correlation; investigate air pollution indices or multi-pollutant models; use standardized metrics of SEP to enhance comparability across studies; and evaluate potentially susceptible populations.

## Background

Cardiovascular disease (CVD) is the leading cause of mortality in the United States and disparities in CVD are substantial and persistent [1, 2]. Both the physical and social environments play a role in the development of this multifactorial disease [2]. Physical environmental exposures, specifically air pollutants, are a major factor in the global burden of disease [3]. Air pollutants linked to CVD and mortality include particulate matter (PM), nitrogen dioxide (NO_2_) and ozone [2]. In particular, PM has been associated with CVD mortality and the development of chronic CVD conditions, including hypertension and ischemic heart disease, as well as acute events like myocardial infarction [4]. Ambient PM levels account for 3.1% of disability-adjusted life years lost and household air pollution levels account for 4.3% in recent global burden of disease estimates [3].

To evaluate the influence of the social environment on health outcomes, researchers utilize material resource- and prestige-based measures to represent an individual’s socioeconomic position (SEP) within a social hierarchy [5, 6]. Lower SEP has been linked to adverse outcomes including reduced life expectancy and higher incidence of CVD [7]. Measurements related to SEP include both access to material resources and psychosocial stress, which have been independently associated with adverse CVD outcomes and markers of risk [8–10]. Access to material resources include characteristics such as income, wealth and educational achievement [6]. Psychosocial stress results when external conditions overwhelm an individual’s ability and resources to manage the negative effects of external stressors [11]. The pathophysiological impacts of psychosocial stress are mediated largely via disruption of an individual’s ability to maintain allostasis, the adaptive processes that maintain homeostasis by producing chemical messengers (such as cortisol and adrenalin) as mediators [12]. Specifically, allostatic imbalance has been shown to result in compromised immune function, wear-and-tear on bodily systems and susceptibility to illness [9, 13].

Previous reviews have summarized the literature on material resources or psychosocial stress as effect modifiers of the relationship between air quality and health [8, 14–19]. They have examined associations related to a range of air pollutants, including PM, ozone and NO_2_, and multiple adverse health outcomes, most frequently general mortality or respiratory effects. Common individual measures of material resource access among these studies include education, household income, and occupation [8, 19]. Education, for example, was found to be a significant effect modifier of the relationship between both sulfate and PM_2.5_ with CVD mortality in the 2000 reanalysis of the Harvard Six Cities and American Cancer Society (ACS) cohorts [20]. Area-level measures are constructed using analogous information to individual measures, e.g., median household income and median education at the census tract or zip code level, or information on properties of the community or physical environment, such as crime statistics, density and type of grocery stores or restaurants and the like [8, 14–19]. Other reviews examined psychosocial stress utilizing measures such as the perceived stress scale (PSS) or allostatic load [8, 18]. This review contributes new information in two major ways: a focus exclusively on CVD, which is warranted given the significant burden of this disease in the United States and across the globe, and inclusion of studies from multiple countries outside of the U.S., Europe and Canada, which provide a more diverse foundation for understanding population differences.

The first purpose of this systematic review is to evaluate research within the past 12 years concerning interactions between air pollution and SEP, expressed specifically as access to material resources and psychosocial stress, focusing on less well-summarized CVD outcomes, exclusively. The second purpose is to identify research gaps within the published literature on this topic and provide recommendations for addressing these gaps in future research.

## Methodology

We identified articles published in peer-reviewed journals within the MEDLINE® database between January 1, 2005 and December 31, 2016. Our systematic review methodology, including a complete list of search terms, is outlined in Table 1 and is based on the PRISMA-P checklist [21]. In addition to restricting publications to the dates above, other eligibility criteria included being written in English, publication in a peer-reviewed journal, a focus on human cardiovascular health effects, and being primary research. In addition, the article must explicitly state, as an aim, the evaluation of the impact of interactions between air pollutants (as the exposure) and indicators of SEP (access to material resources or psychosocial stress) on cardiovascular health. Searches were conducted using PubMed® and included entering an exposure (e.g. particulate matter), a modifier (e.g. education) and an outcome (e.g. inflammation) jointly into the search field.

**Table 1:** Systematic review methodology

Section/Topic	Detail
Objectives	Review articles from the past 12 years that explicitly investigate interactions between air pollution and material resources or between air pollution and stress on cardiovascular events/indicators.
Eligibility Criteria	1) Published from 2005 to 2016
	2) Written in English
	3) Published in a peer-reviewed journal.
	4) Study of human effects
	5) Explicitly seek to evaluate the impact of interactions between air pollution and social factors on cardiovascular outcomes
	6) Primary research study, excluding abstracts, reviews, meta-analyses and op-eds
	7) Any study design
Information sources	MEDLINE® database, accessed via PubMed®
Search strategy	Searches conducted using all combinations of the following three categories of terms connected with the Boolean operator AND:
	1) Exposure to specific pollutants as listed below: “fine particles” (which signify PM less than 2.5 μm in aerodynamic diameter or PM_2.5_), “PM”, “ultrafine particles” (which signify PM less than 0.1 μm in aerodynamic diameter or UFP), “UFP”, “nitrogen dioxide”, “NO_2_”, “particle number”, “PNC”, and “ozone”.
	2) Interaction with or effect modification by socioeconomic position as given by the listing of the following terms: “socioeconomic status”, “SES”, “socioeconomic position”, “SEP”, “income”, “education”, “material resources”, “chronic stress”, “psychological stress”, “psychosocial stress”
	3) Human health outcomes related to cardiovascular health including the following terms: “cardiovascular”, “mortality”, “inflammation”, “blood pressure”
Data management	Records were imported and organized using Excel and EndNote™
Selection process	1) Enter search terms into PubMed
	2) Import identified citations into Excel
	3) Review papers for inclusion criteria– Level 1 (Screening)
	4) Save studies for inclusion
	5) Review references of selected articles for additional studies – Level 2 (Reference Review)
	6) Review newly identified studies for inclusion
	7) Read all included studies for results– Level 3 (Full Review)
Data Items	Information collected on the following types of data from the articles: publication year, language, study design, participants, air pollutant exposure, modifier/susceptibility, health outcomes
Risk of bias	A qualitative assessment of bias was made based on the study design. In the final manuscript we include a statement potential and implications of bias among all papers.
Confidence in cumulative evidence	Qualitative assessment was made based on the number of studies, results, study designs and sample size

We employed a three step system to select articles for inclusion. Level 1 involved review of the abstract, background and methods section of each article to determine inclusion eligibility per our criteria. In Level 2 we reviewed the references of the selected articles to identify additional papers for consideration. Level 3 was a complete review of the selected papers to note additional details and findings. For each article we examined key information such as the indicator(s) of material resources and/or psychosocial stress, pollutants of interest, study population, and findings, including the statistical significance. An insufficient number of articles of similar scope and methods were found to support a meta-analysis. We have included a qualitative assessment of the risk of bias in this review.

## Results

**Fig. 1:**
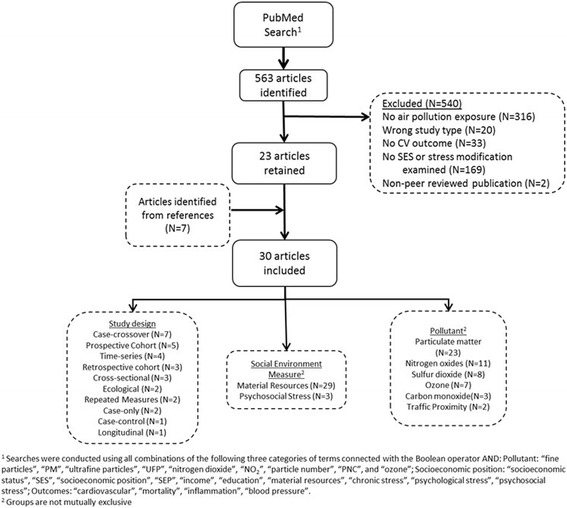
Results of the systematic review

Thirty studies fulfilled the eligibility criteria and are included in this review (Fig. 1). A description of the identified articles is presented in Table 2 and a summary of results is given in Table 3. As part of Level 1 screening, we identified 563 articles for which 23 fit the eligibility criteria. In Level 2, 7 articles were added in reference review. The 30 articles were read and included in Level 3 to identify details for the review.

**Table 2:** Articles examining interactions between air pollution and socioeconomic position on cardiovascular endpoints identified through systematic review, 2005–2016

First Author	Design	Population	Air pollutant exposure(s)	Material resource(s) or psychosocial stress measure(s)	Outcome(s)	Result(s)
Barceló, 2009 [49]	Ecological	Residents of Barcelona, Spain	TSP, PM_10_, NO_2_, CO, SO_2_	Census-tract deprivation index: unemployment, lower educational level, manual workers, temporary workers	Ischemic heart disease mortality	A positive interaction between pollutants and the deprivation index was statistically significant for NO_2_ and ischemic disease mortality in men.
Bravo, 2016 [30]	Case-crossover	Residents of Sau Paulo, Brazil	PM_10_, NO_2_, SO_2_, CO, O_3_	Individual education and area-level SEP index	CVD mortality	Significant positive interaction between pollutants and individual education. Significant inverse interaction between pollutants and SEP index.
Chi, 2016 [40]	Prospective cohort study	Women’s Health Initiative participants from 40 US sites	PM_2.5_	Individual education, family income and occupation. Area-level education, occupation, family income, poverty status, median home value, neighborhood SEP score	CVD event (including MI, stroke, CVD death, cerebrovascular death)	Statistically significant effect modification by neighborhood SEP score. Non-significant higher effect for those with lowest individual income and occupation.
Chiusolo, 2011 [44]	Case-crossover	Adults from 10 Italian cities	NO_2_	Census block group median income and median SEP indicator	Cause-specific mortality	Neither income nor SEP significantly modified the association between NO_2_ and mortality. Significant heterogeneity in the stratum-specific estimates among the cities.
Dragano, 2009 [22]	Cross-sectional	Adults from 3 German cities	Roadway proximity, traffic volume	Individual education and income; Neighborhood unemployment	Coronary artery calcification	Statistically significant effect modification of main effect by education and unemployment among men and modification by income among women.
Finkelstein, 2005 [50]	Prospective cohort	Adults from Hamilton and Burlington, Ontario, Canada	Roadway proximity, TSP and SO_2_	Census tract-level deprivation index: income, education and unemployment	Circulatory disease mortality	Non-significant effect modification by neighborhood deprivation index evident in high traffic areas.
Haley, 2009 [45]	Case-crossover	Residents of New York State with CVD discharge diagnosis	PM_2.5_	Census tract percentage of adults living below poverty level	CVD hospitalizations	No effect modification
Henderson, 2011 [46]	Repeated measures	Canadian population in the southeast corner of British Columbia	PM_10_, smoke	Census tract income quintiles	CVD physician visits and hospitalizations	No main effects of exposures on CVD outcomes (with 2 exceptions). No effect modification
Hicken, 2013 [31]	Cross-sectional	Multi-Ethnic Study of Atherosclerosis (MESA) cohort from 6 U.S. cities	PM_2.5_	Material Resources: Individual education and income and census tract median household income. Stress: Individual chronic stress, depressive symptoms, trait anger, trait anxiety, lack of emotional support	Blood pressure	Non-significant modification showing higher effects among higher education groups and no effect modification by income. No effect modification by stress indicators.
Hicken, 2014 [53]	Repeatedmeasures	Adults in Detroit	PM_2.5_	Stress: Individual environmental stress index, life events index	Blood pressure	Higher effect of PM_2.5_ on blood pressure in people living in Southwest Detroit under high stress.
Hicken, 2016 [48]	Cross-sectional	MESA cohort, 6 U.S. cities	PM_2.5_, NOx	Material Resources: Individual SEP index and census tract racial segregation. Stress: Individual psychosocial adversity	Left ventricular mass index (LVMI), Left ventricular ejection fraction (LVEF)	No effect modification
Kan, 2008 [23]	Time series	Residents of Shanghai, China	PM_10_, SO_2_, NO_2_, and O_3_	Individual education	CVD mortality	Non-significant interaction shows that residents with lower education had an increased risk of CVD mortality compared to those with higher education for all pollutants except O_3_.
Malig, 2009 [24]	Case-crossover	Residents of 15 California counties	Coarse PM	Individual education	Total and CVD mortality	Significant interaction showing that the effect of coarse PM on CVD mortality was higher in those of lower education.
McGuinn, 2016 [42]	Retrospective cohort	CATHGEN Cohort in North Carolina	PM_2.5_	Census block group education, urban/rural	CAD index >23 and MI in the previous year	No effect modification
Medina-Ramon, 2008 [32]	Case only	Residents of 48 U.S. cities	O_3_	Individual education	CVD mortality	No effect modification
Ostro, 2008 [26]	Time series	Residents of California	PM_2.5_	Individual education	CVD mortality	Statistically significant interaction with lower education increasing the effect of PM_2.5_ and its components.
Ostro, 2014 [25]	Longitudinal cohort	Study of Women’s Health Across the Nation (SWAN) cohort	PM_2.5_	Individual education, income, marital status	Continuous CRP; CRP > 3 mg/L; CRP >3 mg/L in high age group	Statistically significant effect modification by income and non-significant effect modification by education.
Qiu, 2015 [47]	Case only	Residents of Hong Kong who died of circulatory/respiratory system diseases	PM_10_, SO_2_, NO_2_, O_3_	Individual employment status	CVD mortality	Significant interaction in that the unemployed were more susceptible to pollution associated mortality for all pollutants except O_3._
Raaschou-Nielsen, 2012 [33]	Prospective cohort	Diet, Cancer and Health study participants in Denmark	NO_2_	Individual education	Mortality due to ischemic heart disease, cardiac rhythm, heart failure, cerebrovascular and other CVD causes	No effect modification
Ren, 2010 [34]	Case-crossover	Population of Eastern Massachusetts	O_3_	Individual education and census tract income and poverty	CVD mortality	No effect modification
Rosenlund, 2008 [52]	Retrospective cohort	Residents of Rome, Italy	NO_2_	Census block group deprivation index	Coronary heart disease mortality and hospitalizations	No effect modification
Rosenlund, 2009 [29]	Case-control	Residents of Stockholm County, Sweden	NO_2_, PM_10_	Individual occupation, education, income and marital status	Fatal and non-fatal MI	Higher effects for low white collar workers and higher income, but no statistically significant effect modification
Son, 2012 [35]	Case-crossover	Residents of Seoul, Korea	PM, NO_2_, SO_2_, CO, O_3_	Individual education, marital status and occupation	CVD mortality	Greater effects for lower education as well as manual occupation and unknown occupation.
Stafoggia, 2014 [27]	Prospective cohort	European Study of Cohorts for Air Pollution Effects (ESCAPE) multi-city participants	PM_2.5_	Individual education and rural/urban residence	Incident stroke	Nonsignificant effect modification by education where the lowest education had highest effect. No effect modification by urban/rural residence.
Wilson, 2007 [41]	Ecological	Residents of central, middle and outer Phoenix, Nevada	PM_2.5_ and PM_10_	Zip code-level income and education	CVD mortality	Lower SEP population may be more susceptible to PM associated mortality, but it is difficult to separate spatial effect.
Winquist, 2012 [43]	Time series	Hospital patients in greater St. Louis MSA	PM_2.5_ and O_3_	Zip code-level poverty	Emergency department visits and hospital admissions for CVD conditions	Higher effect of poverty on O_3_-CVD, all outcomes. Also, poverty on O_3_-CHD, all outcomes. Possible, non-sig differences of poverty on PM_2.5_-CHF relationship
Wong, 2008 [51]	Time series	Residents of Hong Kong, China	PM_10_, SO_2_, NO_2_	Community planning unit social deprivation index	CVD mortality and hospitalizations	Higher mortality from exposure to SO_2_ and NO_2_ for areas with high deprivation index.
Zeka, 2006 [28]	Case-crossover	Residents of 20 U.S. cities	PM_10_	Individual education	CVD mortality	Statistically significant effect modification by education whereby there was a higher PM_10_-associated risk comparing lower to higher education.
Zhang, 2011 [36]	Retrospective cohort	Residents of selected communities in Shenyang, China	PM_10_, SO_2_, NO_2_	Individual education, income and marital status	CVD and cerebrovascular mortality	No effect modification
Zhou, 2014 [37]	Prospective cohort	Adult men from 25 cities in China	TSP (1990–2000), PM_10_ (2000–2006)	Individual education	CVD mortality	No effect modification

Abbreviations: *CAD*, coronary artery disease; *CVD*, cardiovascular disease; *MI*, myocardial infarction; *MSA*, metropolitan statistical area; *SEP*, socioeconomic position

**Table 3:** Evidence of material resources and psychosocial stress as modifiers* of the association between air pollutants and cardiovascular indicators, 2005–2016

	Potential effect modifiers	Effect modification in the expected direction	Effect modification in the opposite direction	No effect modification	Total discrete articles
Material Resources					
Individual measures	Education	Dragano et al. 2009 [22]	Rosenlund et al. 2009 [29]	Chi et al. 2016 [40]	18
		Kan et al. 2008 [23]	Hicken et al. 2013 [31]	Media-Ramon 2008 [32]	
		Malig et al. 2009 [24]		Raaschou-Nielsen et al. 2012 [33]	
		Ostro et al. 2008 [26]		Ren et al. 2010 [34]	
		Ostro et al. 2014 [25]		Rosenlund et al. 2009 [29]	
		Stafoggia et al. 2014 [27]		Zhang et al. 2011 [36]	
		Zeka et al. 2006 [28]		Zhou et al. 2014 [37]	
		Bravo et al. 2016 [30]			
		Son et al. 2012 [35]			
	Income/ Poverty status	Chi et al. 2016 [40]	Rosenlund et al. 2009 [29]	Hicken et al. 2013 [31]	7
		Dragano et al. 2009 [22]		Ostro et al. 2014 [25]	
		Ostro et al. 2014 [25]		Zhang et al. 2011 [36]	
	Occupation/ Unemployment	Qiu et al. 2015 [47]	Chi et al. 2016 [40]		4
		Rosenlund et al. 2009 [29]			
		Son et al. 2012 [35]			
	Deprivation Index			Hicken et al. 2016 [48]	1
	Other	Ostro et al. 2014 [25]		Rosenlund et al. 2009 [29]	5
				Son et al. 2012 [35]	
				Stafoggia et al. 2014 [27]	
				Zhang et al. 2011 [36]	
Area measures	Education	Chi et al. 2016 [40]		McGuinn et al. 2016 [42]	3
		Wilson et al. 2007 [41]			
	Income/ Poverty status	Chi et al. 2016 [40]		Chiusolo et al. 2011 [44]	8
		Wilson et al. 2007 [41]		Haley et al. 2009 [45]	
		Winquist et al. 2012 [43]		Henderson et al. 2011 [46]	
				Hicken et al. 2013 [31]	
				Ren et al. 2010 [34]	
	Occupation/ Unemployment	Dragano et al. 2009 [22]			1
	Deprivation index	Barcelo et al. 2009 [49]	Bravo et al. 2016 [30]	Chiusolo et al. 2011 [44]	8
		Chi et al. 2016 [40]		Hicken et al. 2016 [48]	
		Finkelstein et al. 2005 [50]		Rosenlund et al. 2008 [52]	
		Wong et al. 2008 [51]			
	Other			Hicken et al. 2016 [48]	2
				McGuinn et al. 2016 [42]	
Psychosocial Stress					
Individual measures	chronic stress			Hicken et al. 2013 [31]	1
	depressive symptoms			Hicken et al. 2013 [31]	1
	trait anger			Hicken et al. 2013 [31]	1
	trait anxiety			Hicken et al. 2013 [31]	1
	emotional support			Hicken et al. 2013 [31]	1
	stress index	Hicken et al. 2014 [53]		Hicken et al. 2016 [48]	2
	Total discrete articles	18	4	17	

(*including statistically significant modification and non-significant effect modification)

**Fig. 2:**
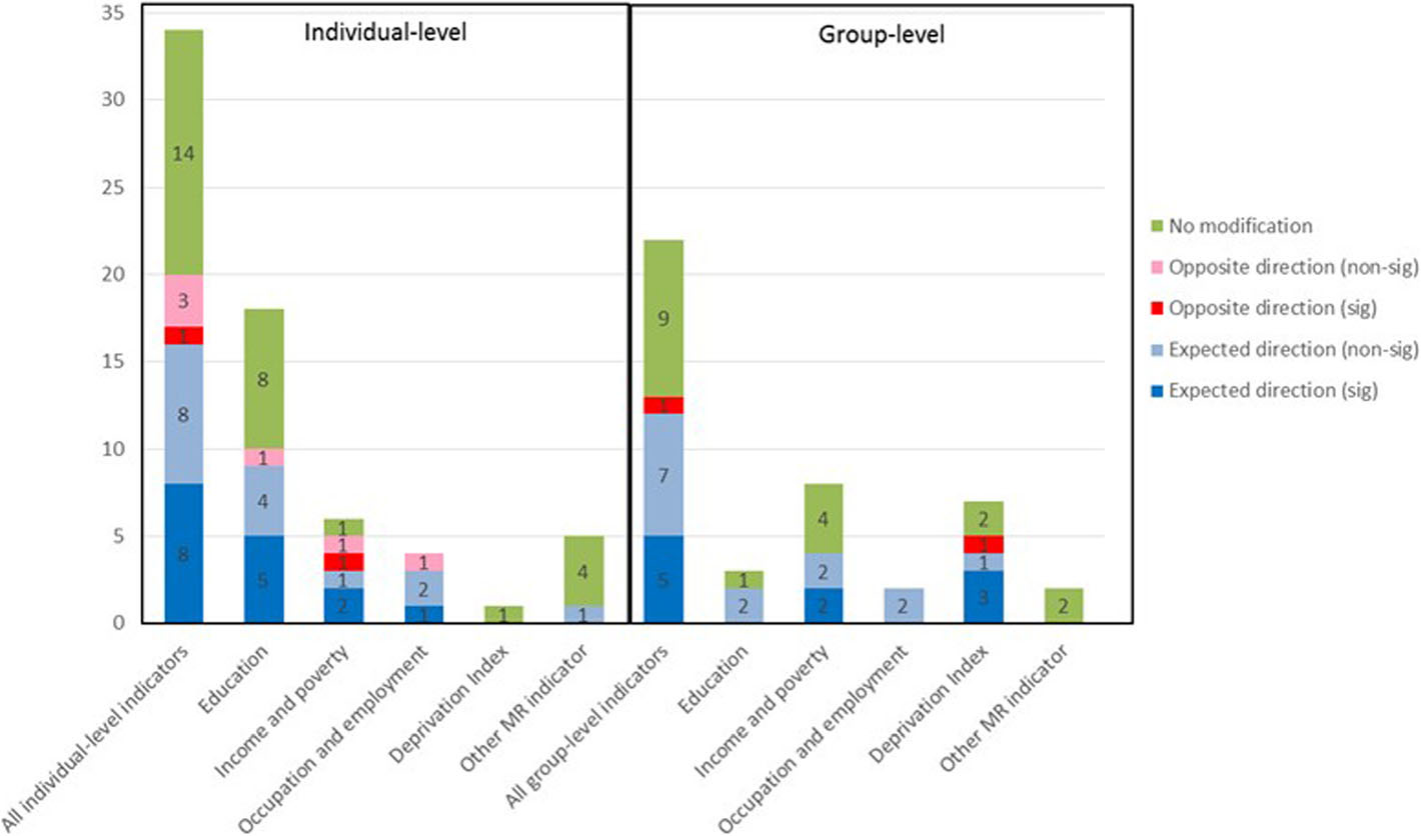
Categorization of material resource indicators among studies (Notes: (1) A single article may examine multiple indicators, (2) Non-sig: not statistically significant effect modification, (3) Sig: statistically significant effect modification)

**Fig. 3:**
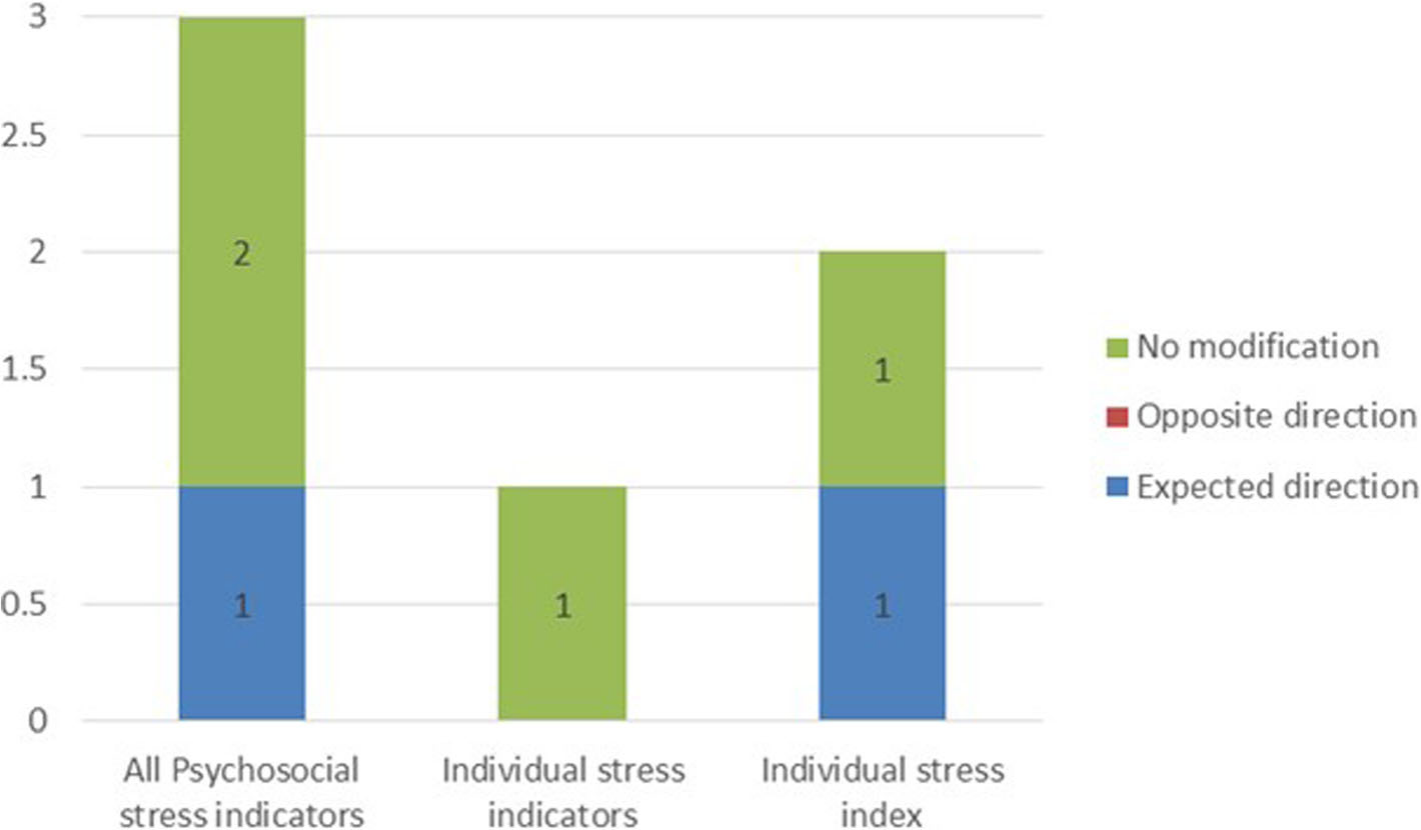
Categorization of psychosocial stress indicators among studies (Note: a single article may examine multiple indicators)

Multiple study designs were used to examine effect modification (Fig. 2). The largest number were case-crossover studies (*N* = 7), followed by prospective cohort studies (*N* = 5) and time-series studies (*N* = 4). Particulate matter was examined in 23 studies, nitrogen oxides (NO_x_) or NO_2_ in 11 studies, SO_2_ in 8 studies and ozone in 7 studies. The majority of articles (*N* = 27) evaluated the impact of material resources only, while two evaluated both material resources and psychosocial stress and one examined effect modification by psychosocial stress only. The hypothesis common among the studies was that lower SEP would be associated with an increased effect of air pollution on CVD. Lower SEP would be indicated by reduced access to material resources and higher levels of psychosocial stress. Here, we initially summarize articles including effect modification by material resources (Fig. 2), then focus on articles examining effect modification by psychosocial stress (Fig. 3). We group the findings for each effect modification analysis into one of three categories: statistically significant effect modification (where there is a clear difference in effect sizes that is statistically significant); non-significant effect modification (there is a different in effect sizes between groups, but the differences are not significant); and no effect modification (there is no difference in effect sizes between groups).

### Education

The material resource measure with the largest number of studies was education. A total of 20 studies evaluated effect modification by this measure at the individual (*N* = 18) or area (*N* = 3) levels [22–37]. Five of the studies that examined individual education identified statistically significant effect modification while 5 others found non-significant differences in effect estimates by education. In a study of 6 California counties by Ostro et al., an interquartile range increase in PM_2.5_ was associated with a significant increase in CVD mortality for individuals with lower education (i.e. without a high school (HS) diploma) [26]. Specifically, 0-day and 3-day lags were associated with a 2–6% increased risk of mortality for those without a HS diploma [26]; results for those with a HS diploma were consistent with no effect. Constituents of PM_2.5_ – including elemental carbon, organic carbon, nitrate and sulfate – also showed similar associations in those with lower education [26].

Zeka et al. conducted a case-crossover study of adults in 20 U.S. cities examining 0- to 3-day lags of PM_10_ [28]. Individual education was grouped into 3 categories: <8 years (low), 8–12 years (medium) or 12 or more years (high). Effect estimates for heart disease mortality were highest for those within the low education stratum and estimates of myocardial infarction (MI) mortality was highest for those in the medium education stratum. Dragano et al. used individual education to examine, in separate models, effect modification of the relationship between proximity to traffic and coronary artery calcification (CAC) in three German cities [22]. The odds ratio was 1.85 (95% CI: 1.16–2.94) for traffic proximity (distance to a major road ≤100 m) and CAC among individuals with 13 or fewer years of education, compared to 1.39 (95% CI: 0.76–2.55) among individuals with 14 or more years of education. In contrast, no material resources interaction with the high traffic exposure-CAC association among women was observed [22]. The interpretation of individual education and income as measures of access to resources may differ between women and men, in part because of gender roles in society, where, for example, women are often paid less for the working the same job as men who have identical educational attainment [38, 39].

Bravo et al. identified statistically significant effect modification by education on the relationship between PM_10_ and CVD mortality. Those with no education had a 3.74% (95% CI: 0.04%, 7.3%) higher risk for CVD death [30]. Malig et al. found a positive interaction between short-term exposure to coarse PM and education dichotomized into less than a HS education compared to a HS diploma or more [24]. The increased risk of cardiovascular mortality was 1.3% (95% CI: 0.1%–2.5% for a 2-day lag) for those with lower education compared to those with higher education. These results did not change after adjusting for PM_2.5_ and restricting to those residing within 10 km of the monitor to reduce exposure misclassification [24]. Investigators conducting a time-series study in Shanghai, China, categorized education into two groups – low, illiterate or primary school and high, middle school or above – and evaluated effect modification of associations between 2-day moving averages of PM_10_, SO_2_, NO_2_, ozone and mortality [23]. Larger, albeit non-statistically significant, CVD mortality estimates were found for short-term exposure to PM_10_, as well as SO_2_ and NO_2_ for those with lower educational attainment compared to those with greater attainment. Associations with ozone were consistent with the null. Ostro et al. estimated a larger effect of PM_2.5_ on inflammation for women of lower education, compared to women of higher education, but not a statistically significant interaction [25]. Higher effect sizes for participants with no education and ≤12 years education compared to participants with >12 years education were identified by Son et al. [35]. The pollutants of interest included PM_10_, SO_2_ and CO and the outcome was CVD mortality.

Chronic exposure to modeled PM_2.5_ was associated with an increased risk of stroke in cohorts from the European Study of Cohorts for Air Pollution Effects (ESCAPE) study [27]. Individual education was categorized into 3 groups: less than primary school, less than secondary school (or equivalent) and greater than a university degree. The main effect was more pronounced in those of lower education, although the effect was not statistically significant. Rosenlund et al. [29] failed to find effect modification of the effect of NO_2_ on MI by education dichotomized as < HS diploma compared to ≥ HS diploma. Hicken et al. [31] found that those in the 90th percentile of education in years had higher and statistically significant effects of PM_2.5_ on systolic blood pressure, pulse pressure and mean arterial pressure. However, being in the 10th percentile showed a lower and non-significant effect. The authors also reported modest correlation between socioeconomic indicators and PM_2.5_ [31].

Additional articles evaluated interactions between several air pollutants and individual education, but did not find evidence of effect modification or found inconsistent results with CVD mortality [32–34, 36, 37] or first CVD event [40]. Medina-Ramón and Schwartz conducted a case-only study of CVD mortality in 48 U.S. cities with ozone as the exposure of interest [32]. Education, dichotomized into less than HS education or HS graduate or more, did not modify the effects of ozone on CVD mortality. A case-crossover study in eastern Massachusetts by Ren et al. evaluated the impact of ozone exposure on CVD mortality and effect modification by individual education categorized into 4 groups (≤8 years, 9–12 years, 13–16 years and ≥17 years) [34]. Although ozone was significantly associated with excess mortality, no differences were found by individual education. Raaschou-Nielsen et al. did not identify effect modification by education groups (<8 years, 8–10 years and >10 years) on the association between NO_2_ and CVD mortality [33]. Similar conclusions were made by Zhang using dichotomized education on associations between PM_10_, SO_2_, NO_2_ on CVD mortality and Chou et al. with dichotomized education (<6 years or ≥6 years) on the associations between PM and CVD mortality [36, 37]. Chi et al. divided education into < HS, HS diploma, some college or associate’s degree and bachelor’s degree or higher. No effect modification was found of the PM_2.5_ and CVD event associations in the cohort of women examined [40].

Only three articles we assessed examined area-level education [40–42]. Chi et al. found higher effect sizes of PM_2.5_ on CVD events for the lowest 2 quartiles of education (represented by percent of adults over 25 with a HS diploma) compared to the highest 2 quartiles. However, there was no statistically significant interaction [40]. A retrospective study of chronic exposure to PM_2.5_ and coronary artery disease (CAD) by McGuinn et al. did not find a difference in effect sizes comparing participants from census block groups with higher percentages of residents with higher education versus lower education block groups. However, the effect for individuals with higher education was statistically significant [42]. Wilson et al. evaluated exposure to 0- to 6-day moving averages of PM_2.5_, PM_10_ and coarse PM (which the authors abbreviated as PM_10–2.5_) and CVD mortality, and effect modification by zipcode-level education [41]. Education was estimated by the percent of the population over 25 with less than a HS diploma. Using an ecological design, mortality rates were compared between central, middle, and outer ring zip codes of Phoenix. Their results indicated that populations with lower material resources may be more susceptible to PM-associated mortality, but results varied by exposure period as well as pollutant and were not significant.

### Income and poverty status

Income and poverty were also used frequently in studies as markers of material resources, with slightly more studies using area-level metrics (*N* = 8) compared to individual level (*N* = 6). We discuss income and poverty in the same section here, but realize that these constructs differ. Dragano et al. evaluated effect modification by individual income as well as education, which we presented previously [22]. A significant interaction was reported only in women, with women in the lowest income stratum having a significantly higher level of CAC associated with pollution exposure compared to women in the highest income stratum. Ostro et al. [25] identified a statistically significant effect of individual income whereby lower-income women had a higher effect of PM_2.5_ on CRP. Hicken et al. also reported statistically significant effect modification of associations between PM_2.5_ and blood pressure in MESA participants’ income, although in the opposite direction, showing a greater burden for those of higher income [31]. Chi et al. noted that women earning less than $20,000 per year had a hazard ratio (HR) of 1.3 (95%CI: 1.12, 1.52) relating PM_2.5_ to CVD events. However, this effect was not significantly different from other groups [40]. Higher effect sizes for higher income participants were also noted by Rosenlund et al.; however, the associations were not statistically significant [29]. The odds ratio (OR) for those of higher income was approximately 3.5 compared to an OR of 2.5 for those of lower income [29]. Another article by Zhang et al. failed to find an interaction between individual income and PM or SO_2_ or NO_2_ [36].

Only two of the eight studies examining area-level poverty, income or wealth reported statistically-significant effect modification, while two others noted differences in effect sizes. Chi et al. identified statistically significant modification of the relationship of PM_2.5_ on CVD events by median home value [40]. Those in the lowest quartile had a HR of 1.4 (95% CI: 1.24, 1.58) compared to 0.87 (95% CI: 0.77, 0.99) in the highest quartile. Percent above poverty was also a statistically significant modifier. Although effect modification was not statistically significant for census tract median income, each decrease in income corresponded to an increase in effect size. [40]. Short-term exposure to PM_2.5_ and O_3_ and impacts on emergency department (ED) visits and hospital admissions were evaluated in the St. Louis area by Winquist et al. [43]. Their results show more pronounced effects of O_3_ on CVD and congestive heart failure (CHF) ED visits and hospitalizations for patients from high poverty areas compared to those from low poverty areas. A case-crossover study conducted in 10 Italian cities by Chiusolo et al. evaluated area-level median income [44]. Analyses of how income modified associations of NO_2_ with CVD mortality varied greatly between cities and pooled results did not reveal a modifying effect of area-level income. Hospitalizations were the focus of a study of susceptibility to PM_2.5_ exposure by census-tract level poverty [45]. The investigators, Haley et al., did not find statistically significant effect modification by poverty, but observed a slight increase in effect estimates for risk in low poverty areas.

A case-crossover study in eastern Massachusetts by Ren et al. evaluated the impact of ozone exposure on CVD mortality and modification by census-tract block group household income and poverty [34]. Although ozone was significantly associated with excess mortality, no differences were found by either of the material resources metrics. Wilson et al. evaluated exposure to 0- to 6-day moving averages of PM_2.5_, PM_10_ and coarse PM and CVD mortality, and effect modification by zipcode-level poverty level [41]. As with education, their results indicated that populations with lower material resources may be more susceptible to PM-associated mortality, but results varied by exposure period as well as pollutant and were nonsignificant. Hicken et al. and Henderson et al. evaluated effect modification of PM associations by income, neither identifying differences in effect sizes or statistically significant effects [31, 46].

### Occupation and unemployment

Five studies evaluated occupation or unemployment, which we grouped together to facilitate discussion. A case-only study by Qiu et al. based in Hong Kong examined all cardiorespiratory deaths over a 10 year period [47]. Occupational status was used to characterize access to material resources, and was dichotomized into “with occupation” or “economically inactive” strata. The authors reported significantly greater effects for “economically inactive” individuals. For this sub-group, a 10 μg/m^3^increase in pollutant concentration averaged over 0–2 day lags was associated with an increase in cardiorespiratory mortality of 1.7% (95% CI: 1.2%–2.1%) for PM less than 10 μm in aerodynamic diameter (PM_10_), 2.0% (95% CI: 1.4%–2.5%) for PM_2.5_, 2.3% (95% CI: 1.7%–2.8%) for NO_2_, and 6.3% (95% CI: 5.2%–7.5%) for sulfur dioxide (SO_2_). In contrast, ozone exhibited the opposite effect, showing a decrease in mortality for an increase in ozone [47]. Rosenlund et al. categorized individual occupation into three groups: blue collar worker, lower-level white collar worker and higher-level white collar worker when evaluating the impact of NO_2_ on fatal MI. In their cohort, effect estimates for lower-level white collar workers were higher than that of higher-white collar workers [29]. Son et al. identified higher effects of most pollutants effects on risk of CVD mortality for manual workers compared to professional workers [35]. The highest effect was a 26.1% increased risk for an IQR increase in NO_2_ [35]. Chi et al. divided individual occupation into 4 groups: managerial/professional, technical/sales/administrative, service/labor and homemaker only. Effect estimates were slightly higher for the first two groups, but occupation did not significantly modify associations between PM_2.5_ and CVD events [40]. Census tract-level occupation (percent adults ≥16 years old with managerial/professional/executive occupation) had higher effects for the lowest quartile, but non-significant effect modification [40]. Dragano et al. noted that for men, the OR for CAC associated with traffic proximity (distance to a major road ≤100 m) was 2.12 (95% CI: 1.22–3.88) in areas with high neighborhood unemployment, whereas the corresponding odds ratios in neighborhoods with medium unemployment was 1.59 (95% CI: 0.80–3.16) and for low unemployment, 1.61 (95% CI: 0.83–3.11) [22].

### Deprivation and socioeconomic indices

Indices of deprivation or socioeconomic status were estimated at the individual level in one article [48] and by area-level in 7 articles [30, 40, 44, 49–52]. Hicken et al. utilized a deprivation index based on individual education, income, paternal education and wealth, but did not observe interaction of this index with PM_2.5_ or NO_x_. Chi et al. built an index of neighborhood SES (NSES) using indicators of education, occupation, family income and poverty. NSES significantly modified associations between PM_2.5_ and CVD events in the all-female cohort. The HR for the most disadvantaged group was 1.39 (95% CI: 1.21, 1.61), which was higher than the HR of 0.90 (95% CI: 0.72, 1.07) for the least disadvantaged group [40]. A study conducted in Barcelona reported a statistically significant interaction between NO_2_ and census-level deprivation on ischemic heart disease mortality among men [49]. Deprivation was defined by an index of census-tract level unemployment, percent lower educational level, percent manual workers and percent temporary workers. The authors also identified non-linear correlations between the deprivation index and pollutants, finding higher NO_2_, CO and SO_2_ co-located in less deprived areas. Finkelstein et al. conducted a prospective study of circulatory mortality and evaluated effect modification by material resources using a deprivation index built from data on income, education and unemployment [50]. In the Finkelstein study, pollution exposure was estimated by: 1) a pollution index combining the concentrations from modeled SO_2_ and total suspended particles (TSP); and 2) proximity measures of distance to roadway (50 m and 100 m). Deprivation, pollution index, and the traffic indicator were all significant predictors of CVD mortality. When deprivation was placed in models with pollution metrics, only the traffic indicator remained significant. However, cumulative hazard curves appeared to show a higher hazard for death for traffic-exposed subjects with high neighborhood deprivation compared to those with low neighborhood deprivation. The authors noted a significant trend of higher levels of TSP, SO_2_, and proximity to roadways in higher deprivation neighborhoods [50]. Wong et al. built a deprivation score for tertiary planning units (TPUs), an administrative unit created for town planning purposes, using six variables including proportion of the population unemployed, a monthly household income below US $250, no schooling, one-person household, never married and subtenancy. The score was divided into tertiles to estimate material resources for the population of Hong Kong [51]. Short-term 0- to 4-day lags of exposure to NO_2_, SO_2_, PM_10_ and ozone were evaluated, and higher effects of NO_2_ and SO_2_ on CVD mortality risk were identified for high deprivation TPUs. The greatest effects were a 2.14% (95% CI: 1.07%–3.21%) excess risk of CVD mortality for a 1-day lag in NO_2_ and a 2.88% (95% CI: 1.35% – 4.43%) excess risk for SO_2_. Bravo et al. utilized an area-level SES index incorporating population density, median age, family income, and housing characteristics in evaluating interaction with multiple air pollutant measurements [30]. For all pollutants except ozone, a statistically significant higher association with CVD mortality was seen for unknown SES compared to low SES. In addition, risks were generally higher for those with medium or high SES compared to low SES [30]. Group-level SES was not associated with effect modification of NO_2_exposure and CVD mortality in two other studies by Rosenlund et al. and Chiusolo et al. [44, 52]. Rosenlund’s measure of SES incorporated education, unemployment rate, occupation, family size, home ownership, crowding and immigration, while Chiusolo et al. did not provide a clear definition.

### Additional material resource measures

Other measures of material resources utilized in studies included marital status, urban/rural residency and racial segregation. Individual marital status was evaluated by the greatest number (*N* = 4) and none of the studies identified effect modification between any pollutant and other CVD mortality or biomarkers of CVD risk [25, 29, 35, 36]. Two studies evaluated urban/rural residency [27, 42]. Participants living in rural areas had a higher increase in the incidence of stroke associated with a 5 μg/m^3^ increase in PM_2.5_ compared to their urban counterparts across all sites in the ESCAPE study [27]. Although the effect size was higher it was not statistically significant. However, residence in a rural area compared to an urban area at the census-tract level was not associated with any difference in the association between PM_2.5_ and MI or CAD index [42]. Hicken et al. evaluated census tract level segregation and did not identify interaction with the effect of PM_2.5_ on left ventricular mass index (LVMI) or left ventricular ejection fraction (LVEF) [48].

### Psychosocial stress

Three articles by Hicken et al. [31, 48, 53] evaluated susceptibility by psychosocial stress and material resources (Figure 3). The first examined blood pressure in the Multi-Ethnic Study of Atherosclerosis (MESA) cohort [31]. The exposure of interest was PM_2.5_ and psychosocial stress was evaluated by measures of chronic stress, depressive symptoms, trait anger, trait anxiety and lack of emotional support. The investigators reported no effect modification by psychosocial stress. The authors also reported modest correlation between psychosocial adversity and PM_2.5_ [31]. The second article found an effect of stress in one of three Detroit communities. The community characterized by high stress had a larger association between PM_2.5_ and blood pressure compared to the other two communities. The association corresponded to a 9.05 mmHg (95%CI: 3.29–14.81) increase in systolic blood pressure for a 10 μg/m^3^ increase in PM_2.5_ [53]. Psychosocial stress was estimated by a sum of high scores on six different scales. Education and poverty-income ratio were both estimated at the individual level. The authors did not find high correlation between material resources and PM_2.5_ [53]. A composite index of psychosocial adversity (comprised of 5 measures including depressive symptoms, trait anxiety, trait anger, chronic stress and lack of emotional support) did not modify the effect of PM_2.5_ on LVMI or LVEF in the MESA study [48].

## Discussion

As highlighted in the current review, considerable and growing interest continues in examining whether socioeconomic factors confer cardiovascular susceptibility to air pollution exposures. Although the literature in this area is still relatively small, gaps and inconsistencies can be identified to inform future research studies. Broadly, in our review, several articles provided evidence of effect modification by individual education, income, occupation and urbanicity. In addition, area-level measures of poverty, unemployment and deprivation were also shown to be effect modifiers. Support for psychosocial stress as an effect modifier was weak; however, only three articles tested this hypothesis. Recommendations can be made to address knowledge gaps and enable more definitive conclusions regarding socioeconomic factors as modifiers for air pollution-related cardiovascular endpoints.

### Selection of measures of SEP

We reviewed articles that employed a variety of individual and area-based measures, or indices, of material resources such as education, income, poverty, unemployment and urban/rural residence. Psychosocial measures included chronic stress, depression, anxiety and emotional support. The choice of measures representing SEP depends on the hypothesis of how SEP is related to health as well as the availability of the measure in the research dataset [54]. Each measure has an underlying construct and potential mechanism of effect that should be stated explicitly. Notably, individual and area-level SEP may differ, conceptually, in reflecting mechanisms within which effect modification occurs, although both variables capture elements of social condition.

While the majority of studies identified effect modification by material resources in the expected direction, other findings were opposite of the hypothesized direction. This could be a function of the measures themselves and mechanisms by which they have their effect. Observation of statistically significant effect modification may signify that the indicator is a good measure of access to material resources important to health. However, alternative explanations include: less error in a particular measure (i.e., reduced Type I error) thereby making a significant result more likely if an association exists; or, the measure is highly correlated with the true causal modifier and is thereby confounded. An index may better capture area-level access to material resources by incorporating multiple aspects of this construct into one measure. However, an index may also increase error if its components are measured with error. Area-level factors can also affect individual conditions; for example, income inequality can alter the effect of individual income on health outcomes [55]. Most studies do not examine both levels jointly, which precludes assessment of these relationships. However, multilevel studies have the ability to simultaneously evaluate how individual and area-level factors impact individual health status.

### Recommendation

Clear, standardized definitions of metrics facilitate comparison across and interpretation of studies. However, using identical measures across studies also has limitations, given the specific context of each study. Measures that capture SEP well for one population may not do so for another, but studies conducted in similar populations could use similar measures. Also, in addition to individual or area-level measures of SEP, proximal and distal factors that accumulate across the life course could modify air pollution associations with health, and should be evaluated to the extent possible [16, 56]. Including metrics at multiple levels in the same model is now possible using new analytical techniques that can help uncover the variance explained by multiple predictors.

### Air pollutants of concern

**Fig. 4:**
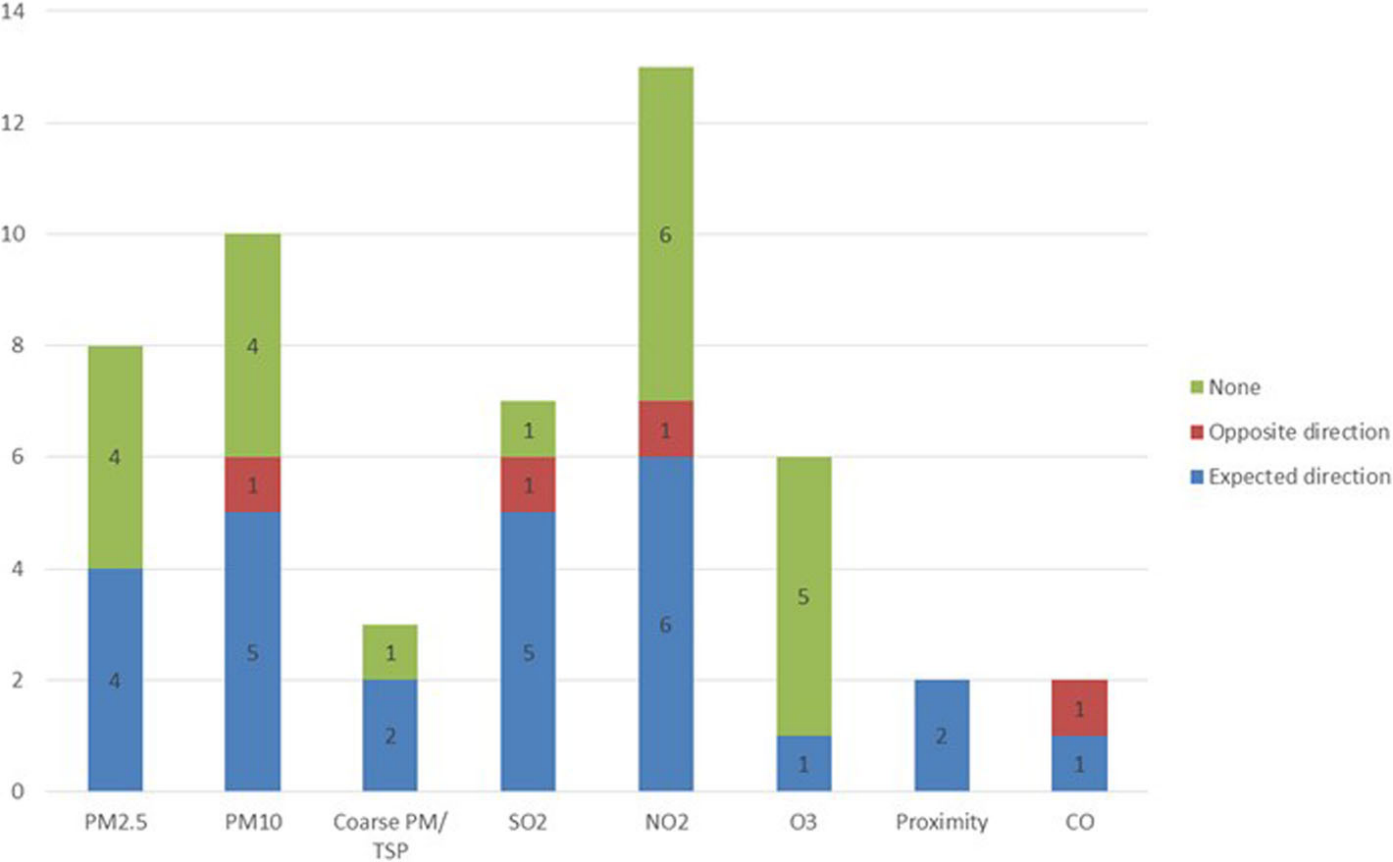
Differences in effect estimates according to pollutant in the reviewed studies (for articles examining material resources only)

Our review sought to include studies of multiple air pollutants. Results according to pollutant are given in Fig. 4. Only one article examining ozone as the pollutant of interest in our review identified effect modification of associations [43], despite the fact that ozone was a significant predictor of CVD outcomes in some of these articles [32, 34, 35]. By contrast, several articles found that material resources modified associations of CVD health outcomes with other pollutants, including NO_2_ and PM. A prior review reported that SEP modified the association between ozone and total mortality, respiratory and respiratory hospitalizations [17]. The association between ozone exposures and cardiovascular health may not be as strong as for respiratory outcomes. A recent experimental study did not find associations between low-level ozone exposure and cardiovascular function or systemic inflammation [57].

### Recommendation

Because many air pollutants co-vary, future studies may more fully test for effect modification using multi-pollutant models, pollutant indices or approaches for studying mixtures.

### Correlations between air pollution and SEP

A critical barrier to understanding CVD disparities is the interrelated nature of physical and socioeconomic conditions [8]. Correlation between air pollutants and SEP are not typically reported in studies. In this review, only a handful of the identified articles explicitly evaluated spatial correlation between these covariates [22, 27, 31, 49, 50, 53]. Staffogia et al. examined spatial autocorrelation using a “frailty” model, and did not identify any differences in effects [27]. In two articles by Hicken et al., the authors evaluated correlation between PM_2.5_ and both material resource and psychosocial stress measures using variance inflation factors (VIF). The VIF provides an estimate of multicollinearity between variables in an ordinary least squares regression [58]. The average VIF for the MESA study variables was 4.49 and the maximum VIF factor was 3.42 in the study based in Detroit [31, 53]. A weakness of the VIF is that there is no clear boundary to designate multicollinearity [58]. The threshold to distinguish multicollinearity was 10 in the MESA article and 5 in the Detroit article. For the MESA study, the average VIF comparing SEP and PM_2.5_ was 4.49 for all cities, suggesting that one or more city-specific VIFs may be above the VIF threshold of 5. Thus, depending on the threshold, multicollinearity may be a source of concern in these articles. Barceló et al. identified moderate correlations between deprivation and SO_2_ (*r* = −0.347), NO_2_ (*r* = −0.329), CO (*r* = −0.440) in Barcelona [49]. Contrary to expectation, low deprivation areas were those with the highest reported pollutant concentrations. Finkelstein reported higher levels of TSP and SO_2_ and closer proximity to roadway in high deprivation neighborhoods, with a significant trend in Hamilton, Ontario [50]. Dragano et al. tabulated traffic pollutant exposure and material resource measures. For males, the highest exposure was shown for lower levels of each material resource metric (i.e. neighborhood unemployment, individual income and individual education) [22]. The trend was the same for individual income and education in women; however, low neighborhood unemployment correlated with high exposure (i.e. distance to roadway ≤100 m or ≤50 m). All articles found some measure of correlation between material resources and pollution, and a conclusion of significant correlation was stated for some articles [49, 50]. In some cases, higher pollutant concentrations were linked to neighborhoods or individuals of higher material resources [22, 49], while the opposite was true for other areas [22, 59]. This may be a function of particular spatial characteristics of residential areas, transportation networks and industrial areas in different urban regions. The picture was even more complex when male and female participants were evaluated separately [22].

### Recommendation

Greater attention should be paid to examining correlation due to the possibility for confounding and bias, the impact on interpretation of results, as well as to aid understanding of underlying fundamental causes [18].

### Diversity of populations

Another key aspect to explore in depth is the difference within and between various racial/ethnic populations. The largest number of articles, 16, were done in North America; 7 in Europe, 6 in Asia and 1 in South America. Some of the sample populations were racially/ethnically diverse while others were less so. Measures of SEP are known to reflect different access to resources according to racial/ethnic group. For example, the benefits associated with a specific level of education or income have been found to be lower for blacks compared to whites, and perhaps other groups [6]. Although we did not do so, other articles utilize race/ethnicity as an indicator of SEP [24, 25, 48].

### Recommendation

To better understand this phenomenon, studies should be done in diverse populations with sufficient numbers of racial/ethnic minorities to evaluate differences.

The scope of our review is narrowly defined to identify papers with a specific focus on examining modification and provide a substantive discussion of effect modification, underlying constructs, measurement tools and comparison with other studies. The review methodology followed the PRISMA-P checklist and we are confident that we have captured relevant papers with a primary aim of exploring effect modification by material resources or stress [21]. We understand that the narrow scope of our review may exclude some articles that examine effect modification as a secondary aim. For example, Miller et al. identified non-significant trends in the Women’s Health Initiative whereby estimated effects of PM_2.5_ on cardiovascular events increased as education and income decreased [60]. Atkinson et al. noted the opposite effect for small area deprivation which showed non-significant increases in effect between air pollution (PM_10_, SO_2_, NO_2_) and cardiovascular disease based on higher affluence [61]. There was no discernible effect modification by deprivation when ozone was the exposure [61]. Also, several other articles reporting on large studies such as ESCAPE, NIH-AARP and DUELS did not identify effect modification by indicators including education, urban/rural residence, or SEP index [62–66]. Even so, we feel that our narrow scope provides a balanced view of the evidence by including articles with a specific focus on investigating modification and reporting results, whether significant or not. Other limitations of our systematic review include our focus on recent articles. Articles published prior to our cutoff date may have been informative to our assessment. However, prior reviews have included many of those studies. In addition, due to the variety of modifiers utilized by the studies, an insufficient number used similar measures to conduct a meta-analysis. In addition, we did not assess publication bias in this review. Therefore, evidence of effect modification may be more nuanced than what is presented here. Lastly, our review is not confined to studies in the U.S., leading to a broader assessment of evidence from populations across the globe.

## Conclusion

This systematic review is the first of its kind to summarize the literature related to air pollution-induced CVD and susceptibility by material resources and psychosocial stress. Interest in understanding these types of interactions is rapidly growing. Broadly, our review identified articles showing that associations between air pollutants including PM and select gases and CVD endpoints were modified by multiple, separate indicators of material resources. Support for CVD susceptibility to air pollution by psychosocial stress was weak, perhaps due to the small number of articles addressing this hypothesis.

Certain considerations can be made to ensure a more focused approach to better assess effect modification both within and between studies. Further, studies with new approaches are needed to develop a more robust and comparable set of evidence regarding how air pollution and SEP may together influence cardiovascular health. Specifically, taking action to establish standardized metrics, incorporate diverse populations and utilize multi-pollutant models or air pollution indices is advisable to strengthen future study designs. In addition, information regarding correlation between air pollutant and socioeconomic indicators is necessary to reduce potential bias. It is also advisable to examine both individual and area-level measures of material resources and psychosocial stress so that independent and joint effects can be evaluated. Future studies can make greater gains in testing associations and mechanisms by enacting some of the changes recommended here.

## Abbreviations

CAC: Coronary artery calcification
CAD: Coronary artery disease
CI: Confidence interval
CO: Carbon monoxide
CVD: Cardiovascular disease
HR: Hazard ratio
HS: High school
MI: Myocardial infarction
NO_2_: Nitrogen dioxide
O_3_: Ozone
OR: Odds ratio
PM_10_: Particulate matter less than 10 μm in aerodynamic diameter
PM_2.5_: Particulate matter less than 2.5 μm in aerodynamic diameter
SEP: Socioeconomic position
SES: Socioeconomic status
SO_2_: Sulfur dioxide
TPU: Tertiary planning unit
TSP: Total suspended particles
VIF: Variance inflation factor
